# A robust bacterial assay for high-throughput screening of human 4-hydroxyphenylpyruvate dioxygenase inhibitors

**DOI:** 10.1038/s41598-019-50533-1

**Published:** 2019-10-02

**Authors:** Jessie Neuckermans, Alan Mertens, Dinja De Win, Ulrich Schwaneberg, Joery De Kock

**Affiliations:** 1 Department of In Vitro Toxicology and Dermato-cosmetology, Faculty of Medicine and Pharmacy, Vrije Universiteit Brussel, Laarbeeklaan 103, B-1090 Brussels, Belgium; 2 Lehrstuhl für Biotechnologie, RWTH Aachen University, Worringerweg 3, 52074 Aachen, Germany

**Keywords:** High-throughput screening, Expression systems

## Abstract

Hereditary tyrosinemia type 1 (HT1) and alkaptonuria (AKU) are inherited metabolic disorders caused by defective enzymes involved in tyrosine catabolism. Nitisinone, an ex-herbicide and member of the β-triketone family, is therapeutically applied to prevent accumulation of toxic metabolites in patients by inhibiting the enzyme 4-hydroxyphenylpyruvate dioxygenase (HPD). Here, we developed a colorimetric bacterial whole-cell screening system that allows quantifying the inhibitory effects of human HPD inhibitors in a high-throughput and a robust fashion. The principle of our screening system is based on the degradation of tyrosine through 4-hydroxyphenylpyruvate into homogentisate by human HPD expressed in *E. coli* and subsequent production of a soluble melanin-like pigment. With the aim to optimise the assay, we tested different *E. coli* strains, expression and reaction temperatures, and time-points for supplementing the substrate. We found that in our assay the addition of prototypical β-triketone HPD inhibitors decreases pigment production in a dose-dependent manner with increasing inhibitor concentrations. In addition, plate uniformity, signal variability and spatial uniformity assessment showed that we have developed a robust high-throughput screening assay that is simple to use, cost-effective and enables identification and evaluation of novel therapeutic human HPD inhibitors for the treatment of tyrosine-related metabolic disorders.

## Introduction

Tyrosine-related inherited metabolic disorders (TIMD) are caused by a single dysfunctional enzyme of the tyrosine degradation pathway. These disorders can be fatal and/or chronically debilitating and include, among other, two autosomal recessive disorders, i.e. hereditary tyrosinemia type 1 (HT1 – OMIM #276700) and alkaptonuria (AKU – OMIM #203500).

HT1 is the most severe TIMD with an overall incidence of 1 in 100.000 newborns worldwide. However, significantly larger numbers of new patients are located in the province of Quebec (Canada), Scandinavia, Turkey and India. One deficient enzyme, the terminal enzyme of the tyrosine catabolic pathway, i.e. fumarylacetoacetate hydrolase (FAH, EC. 3.7.1.2), underlies HT1. Clinical symptoms of HT1 are heterogeneous and comprise hepatic dysfunction, renal tubular failure and neurological crises. This is due to the formation and accumulation of toxic intermediates (i.e. fumarylacetoacetate, maleylacetoacetate and succinylacetone) upstream of the deficient enzyme. Without proper treatment, most HT1 patients die at their infancy^1–3^.

AKU is a serious, multisystem disorder, for which there is momentarily no effective treatment, and affects approximately 1 in 250.000 people, although a higher birth prevalence of around 1 in 19.000 is observed in the Dominican Republic and Slovakia. This disease arises from a deficient homogentisate 1,2-dioxygenase (HGD) enzyme, causing increased levels of plasma HGA and urinary excretion due to the hampered metabolisation of homogentisic acid (HGA). In a process called ochronosis, after the excess HGA is deposited in the collagenous tissues, HGA polymerises in the cartilages of the joints^4^. As AKU is mainly a chronic and progressive disorder with heterogeneous symptoms like spondyloarthropathy, rupture of ligaments, muscles and/or tendons, valvular heart disease and renal, prostate or gall bladder stones, acute fatal metabolic as well as non-metabolic complications can also occur^3,5–7^.

Since 1992, the quality and quantity of life of TIMD patients improved remarkably due to the former orphan drug and ex-herbicide, nitisinone [2-(2-nitro-4-trifluoromethylbenzoyl)-1, 3-cyclohexanedione; NTBC]. Nitisinone reversibly inhibits an enzyme upstream of FAH, i.e. 4-hydroxyphenylpyruvate dioxygenase (HPD, EC 1.13.11.27), an iron-(II)-dependent, non-haem, oxygenase and thereby prevents the formation of HGA out of 4-hydroxyphenylpyruvate (HPP), which comprises the second step in the metabolism of tyrosine. By inhibiting HPD, the amassing of toxic intermediates is obviated and the HT1 phenotype is alleviated^8–10^. Nitisinone rescues HT1 patients from severe illness or even death, and, notwithstanding its unknown value in AKU, this drug is currently the only HGA abating therapy with a potential to alter the evolution of AKU. However, it also leads to hypertyrosinemia which requires lifelong dietary adjustment in order to reduce the chance to develop ocular, cutaneous, and neurological complications^1,3,5–7,9^.

Nitisinone, a member of the β-triketone family of herbicides, is a potent competitive inhibitor of HPD that binds reversibly to the enzyme in a dose- and time-dependent manner. Nitisinone and other members of this family, including mesotrione, sulcotrione and tembotrione, also have agricultural purposes and are used as bleaching weed-killers disrupting pigment production and photosynthesis by inhibiting HPD enzymes in plants^2,9,11^. As nitisinone is a chemically unstable and expensive drug and no alternative therapeutic options are currently approved for clinical use of HT1 and AKU, there is a need for new potent inhibitors with better physicochemical features and toxicological profile^9,12^. Furthermore, since the human toxicological evaluation of nitisinone is inadequate, it makes new HPD inhibitors, with a more reassuring IC_50_ and LD_50_ profile than nitisinone, requested^13^. In addition, human-specific HPD inhibitors are desired over pan-species inhibitors to prevent metabolic perturbations in the patient’s microbiome^14^. A major impediment is finding new HPD inhibitors as the output of drug development is poor despite the large allocated budgets and time investment of the pharmaceutical industry. This problem – in combination with the worldwide competitive pressure – forced the industry to also extensively explore the repositioning of existing drugs^15^. In both cases, it is extremely important to have robust and convenient high-throughput screening (HTS) assays.

In the context of *de novo* drug discovery or drug repositioning for the treatment of TIMD, we aimed to develop a straightforward, colorimetric, and inexpensive HTS system in bacteria which depends on the activity of human HPD. Such a screening system will allow to identify new and human-specific HPD inhibitors for the development of TIMD therapies since targeting HPD for herbicidal purposes is mainly the focus nowadays^16,17^. Previously, a bioassay for the rapid detection of HPD inhibitors as potential herbicides for plant HPD^18^ and an analytical HPLC-method for the evaluation of HGA in the absence or presence of an HPD inhibitor have been developed^19,20^. Although these novel screening systems are useful, they rely on the use of plant HPD enzymes and therefore are not applicable to identify human-specific HPD inhibitors. Furthermore, existing HTS assays, using human HPD, pinpoint on a coupled enzyme method involving HGD and HPD, which is time-consuming, laborious and complex^16,17,21,22^. However, a robust and simple whole-cell bioassay involving only human HPD is not yet available, but is of high relevance for TIMD-related drug discovery and repositioning.

**Figure 1:**
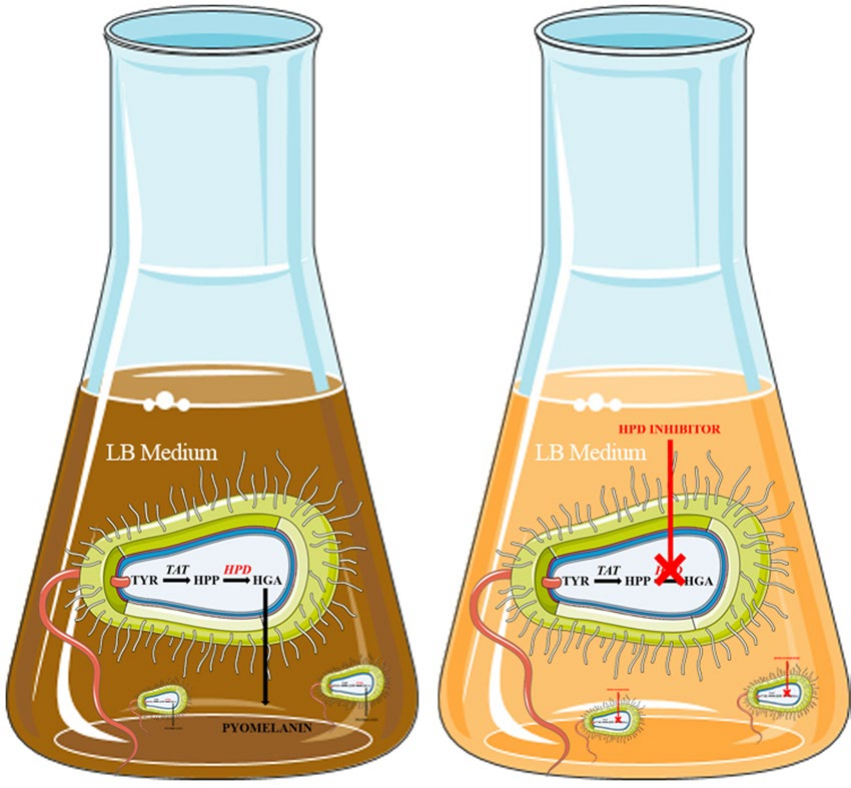
Due to the presence of transaminases, *E. coli* can convert TYR to HPP, which will be further metabolised into HGA by the expressed recombinant human HPD enzyme. Subsequently, due to the absence of HGD in *E. coli*, HGA will auto-oxidise and self-polymerise to produce a melanin-like ochronotic pigment, also known as pyomelanin which will exhibit a characteristic brown coloration. In the presence of an HPD-inhibitor, HPD activity is blocked and this ochronosis process will be reduced or prevented. This figure was produced, in part, by using Servier Medical Art.

With the aim to generate an HTS method, that evaluates the activity of chemical inhibitors, based on human HPD, *Escherichia coli* (*E. coli*) is the preferred organism to use as a manufacturer for the production of recombinant enzymes or proteins, because genetic engineering as well as HTS setups in microtiter plates are well-established^23,24^. Furthermore, *E. coli* can be cultured inexpensively, have ideal growth kinetics, and transformation with exogenous DNA and expression is fast and easy^18,25,26^. Due to the natural presence of transaminases, *E. coli* are also capable of easily converting L-tyrosine (TYR) to HPP, the first step in the metabolism of TYR, which is then further metabolised into HGA by the ectopically expressed human HPD enzyme. Subsequently, accumulated HGA will auto-oxidise to benzoquinoneacetic acid due to the absence of HGD in *E. coli* and self-polymerise to produce a melanin-like ochronotic pigment. The melanin-like ochronotic pigment is also known as pyomelanin and exhibits a characteristic brown colour. Opposed to currently existing screening assays, our newly developed colorimetric bioassay is based on the quantification of pyomelanin, derived from TYR under aerobic and physiological conditions (pH, temperature) relevant to humans. In the presence of an HPD-inhibitor this ochronosis process will be reduced or even prevented when HPD activity is blocked (Fig. 1)^18,27,28^. Altogether, the main goal of this study was to develop a bacterial whole-cell HTS assay based on human HPD that allows to analyse the inhibitory capacity of new potent and/or specific inhibitors of the human HPD enzyme and thereby to evaluate their therapeutic potential.

## Results

### Optimisation of human HPD expression from *E. coli*

**Figure 2:**
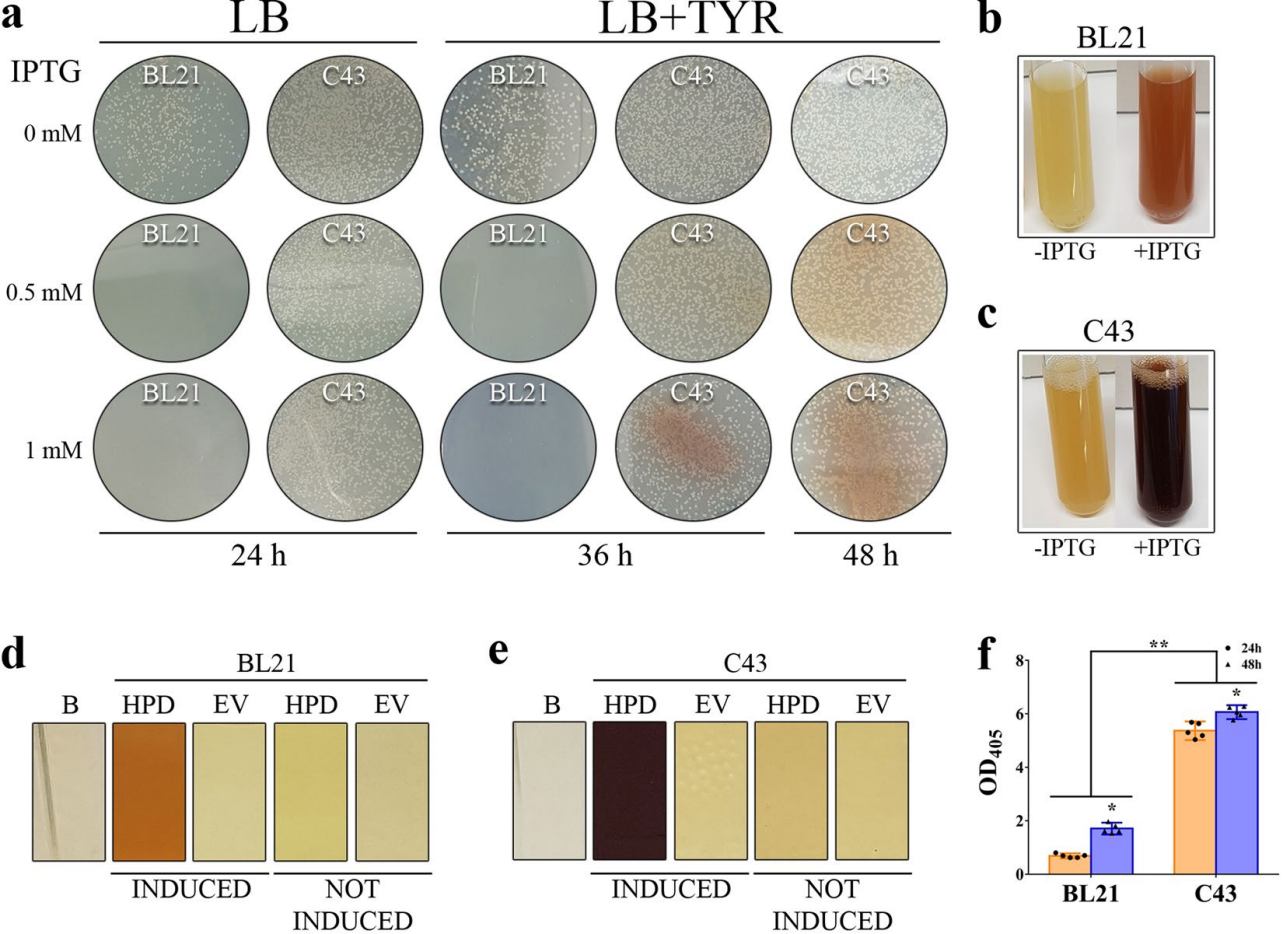
(**a**) On LB_KANA_-agar plates with IPTG induction (0.5 mM or 1 mM), growth of *E. coli* BL21 gold (DE3), expressing human HPD, was absent whereas BL21 gold (DE3) EV control plates showed normal bacterial growth (not shown). This indicates the toxicity of the HPD protein for this strain. This is not the case for C43 (DE3) for which growth and expression is unaffected even when higher concentrations of IPTG were used. Pyomelanin production was detected in the C43 (DE3) LB_KANA_-agar plates after induction, which confirms successful expression of active recombinant human HPD enzyme by C43 (DE3) *E. coli*. (**b**–**e**) LB_KANA_ liquid medium was inoculated with C43 (DE3) or BL21 gold (DE3), uninduced or induced with 1 mM IPTG, supplemented with 0.75 mg/mL TYR, to compare protein expression and activity in liquid cultures. *E. coli* BL21 gold (DE3) and C43 (DE3) liquid cultures clearly showed the production of pyomelanin pigment 24 h after induction, whereas this was not the case for the empty vector and not induced control cultures. (**f**) Quantitative measurement 24 hours after induction shows that pyomelanin production by BL21 gold (DE3), expressing human HPD, lead to an OD_405_ of 0.688 ± 0.074, whereas C43 (DE3) achieved significantly higher pyomelanin levels as shown by an OD_405_ of 5.370 ± 0.281. This resulted in a 7.8-fold higher OD_405_ for C43 (DE3) compared to BL21 gold (DE3). 48 hours after induction, OD_405_ for C43 (DE3) was 6.064 ± 0.209 and reached 1.708 ± 0.178 for BL21 gold (DE3) which is still 3.5-fold lower compared to C43 (DE3).

First subcloning of cDNA of wild type human HPD in a pET42b(+) vector resulted in an expression vector that was transformed by heat shock in two different *E. coli* strains, B21 gold (DE3) and its derivative C43 (DE3), where after they were compared to identify the most optimal strain for reliable expression of the human HPD enzyme. Lysogeny Broth (LB) with Kanamycin (LB_KANA_)-agar plates, containing isopropyl-β-D-thiogalactopyranoside (IPTG) and TYR, were inoculated. On LB_KANA_-agar plates with IPTG induction, i.e. 0.5 mM and 1 mM IPTG, growth of *E. coli* BL21 gold (DE3), expressing human HPD, was absent as seen in Fig. 2a whereas BL21 gold (DE3) empty vector control plates showed normal bacterial growth (data not shown). This indicates that the produced protein is toxic for this strain. This is not the case for C43 (DE3) for which growth and expression is unaffected even when higher concentrations of IPTG were used. We also detected pyomelanin production, secretion and accumulation in the C43 (DE3) LB_KANA_-agar plates after IPTG induction, which confirms the successful expression of active recombinant human HPD enzyme by C43 (DE3) *E. coli* (Fig. 2a).

Next, LB_KANA_ medium was inoculated with C43 (DE3) or BL21 gold (DE3), uninduced or induced with 1 mM IPTG, supplemented with 0.75 mg/mL TYR, in order to compare protein expression and activity in liquid cultures. *E. coli* BL21 gold (DE3) (Fig. 2b,d) and C43 (DE3) (Fig. 2c,e) liquid cultures clearly showed the production of pyomelanin pigment 24 h after induction, whereas this was not the case for the empty vector and not induced control cultures (Fig. 2b–e). Quantitative measurement 24 hours after induction showed that pyomelanin production by BL21 gold (DE3), expressing human HPD, lead to an OD_405_ of 0.688 ± 0.074, whereas C43 (DE3) achieved significantly higher pyomelanin levels as shown by an OD_405_ of 5.370 ± 0.281 (Fig. 2f). This resulted in a 7.8-fold higher OD_405_ for C43 (DE3) compared to BL21 gold (DE3). Forty-eight hours after induction, the acquired OD_405_ for C43 (DE3), 6.064 ± 0.209, was still 3.5-fold higher compared to BL21 gold (DE3), 1.708 ± 0.178 (Fig. 2f). These results demonstrate that *E. coli* C43 (DE3) is the preferential strain to efficiently express the human HPD enzyme for successive experiments.

**Figure 3:**
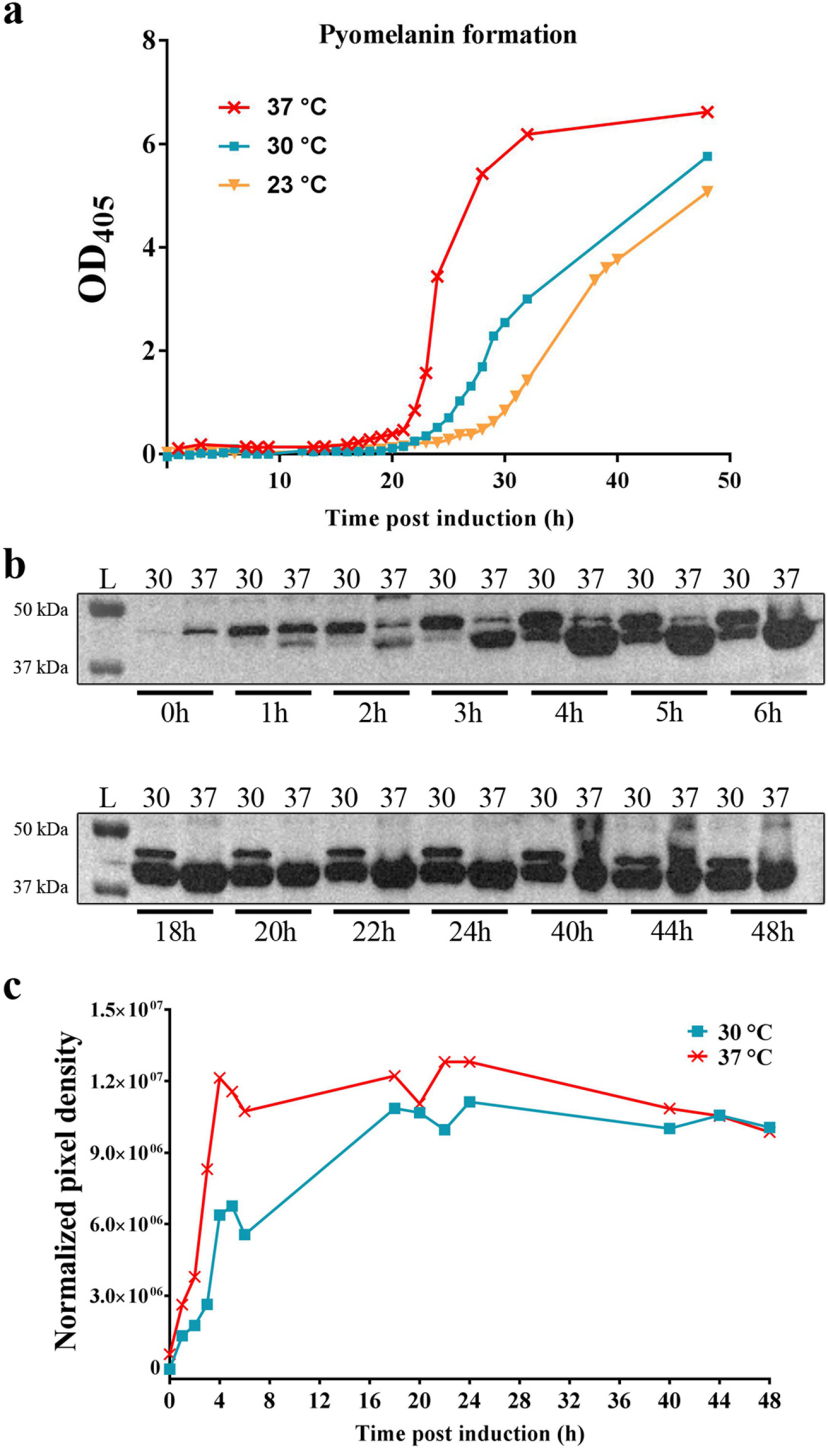
(**a**) Protein induction with C43 (DE3) cells containing pET42b (+) – HPD was assessed at different temperatures (23 °C, 30 °C and 37 °C). Quantification measurements at OD_405_ in the presence of TYR showed that during the first 15 h ochronotic pigment formation was undetectable whereas the onset of pyomelanin formation was the fastest and the production the highest at 37 °C and the lowest at 23 °C. 24 hours after induction the OD_405_ reached its plateau at 37 °C. (**b**–**c**) Western blot analyses of the pellet fraction were performed for human HPD production by C43 (DE3) at 30 °C and 37 °C. At circa 45 kDa a prominent protein band was observed after IPTG induction in the pellet fraction for the two assessed temperatures. Western blot analysis (**b**) and pixel density comparison (**c**), showed that the human HPD enzyme, represented by the lower band at ~45 kDa, was already weakly expressed one hour after induction with 1 mM IPTG at 37 °C, but not at 30 °C. 4 hours after induction the protein was highly expressed at 37 °C, but only moderately at 30 °C. Uncropped blots are available as Supplementary Information in Supplementary Fig. 1.

To define the optimal reaction temperature, we performed protein induction assays with C43 (DE3) cells containing pET42b(+) – HPD pre-grown at 30 °C in LB_KANA_ medium. When OD_600_ reached 0.6–0.8, protein expression was induced with 1 mM IPTG and assessed at different temperatures (23 °C, 30 °C and 37 °C). Quantification measurements at OD_405_ for different incubation temperatures in the presence of TYR showed that during the first 15 h of induced HPD expression ochronotic pigment formation was undetectable whereas the onset of pyomelanin formation was the fastest and the production the highest at 37 °C and the lowest at 23 °C (Fig. 3a). Therefore, for subsequent experiments, the 23 °C condition was not further evaluated. Next, western blot analyses of the supernatant and pellet fraction were performed for human HPD production by C43 (DE3) at 30 °C and 37 °C. At the expected molecular mass for HPD, circa 45 kDa, an overproduced protein band was observed after IPTG induction in the pellet fraction (Fig. 3b) for the two assessed temperatures, but not in the supernatant fraction (data not shown). Western Blot analysis showed that the human HPD enzyme, represented by the lower band at ~45 kDa, was already weakly expressed one hour after induction with 1 mM IPTG at 37 °C, but not at 30 °C. Four hours after induction the protein was already highly expressed at 37 °C, but only moderately expressed at 30 °C. As such, it was observed that high protein expression was much later achieved at 30 °C compared to 37 °C (Fig. 3b,c). These results illustrate that the activity of the human HPD enzyme should preferentially be monitored at 37 °C and at the earliest of 17 hours after induction in order to allow the completion of the auto-oxidation and self-polymerisation process of HGA into the melanin-like ochronotic pigment. Therefore, quantitative measurement of pyomelanin formation using OD_405_ was performed in subsequent experiments 24 hours after induction as from that time point the OD_405_ has reached its plateau (Fig. 3a).

### Dose response inhibition curves

In order to develop a robust HTS bioassay to screen for new human HPD inhibitors, the effect of nitisinone on the recombinant C43 (DE3) HPD strain was examined. *E. coli* human HPD expressing cells were cultured in test tubes, induced with 1 mM IPTG and incubated in LB_KANA_ medium supplemented with 0.75 mg/mL TYR in the presence of 100 µM nitisinone. The addition of this HPD inhibitor decreased pyomelanin formation, leading to a final OD_405_ of 0.51 ± 0.14 after 24 h of incubation. When no nitisinone was added OD_405_ reached 4.27 ± 1.50.

**Figure 4:**
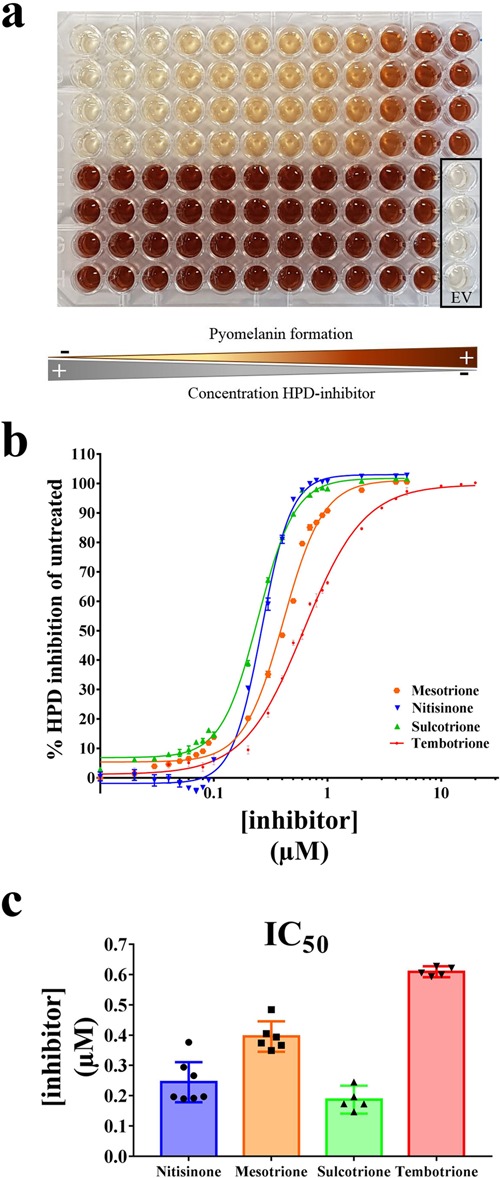
(**a**) Formation of the pyomelanin pigment was observed for *E. coli* HPD. Adding one of the prototypical β-triketone inhibitors decreased pyomelanin formation in a dose-dependent manner with increasing inhibitor concentrations (shown for sulcotrione 0–5 µM). The EV obtained an OD_405_ (OD_405-EV_) of 0.38 ± 0.04 and was used as a negative control. (**b**) The percentage of inhibition was calculated following Eq. 1 and Eq. 2. The calibration curves for the β-triketone inhibitors were fitted by the sigmoidal logistic four-parameter equations. (**c**) The IC_50_ values calculated for each of the HPD inhibitors were 0.396 ± 0.048 µM (mesotrione), 0.244 ± 0.071 µM (nitisinone), 0.187 ± 0.037 µM (sulcotrione) and 0.610 ± 0.015 µM (tembotrione).

Next, we miniaturised the assay into a 96-well microplate format to allow HTS evaluation of HPD inhibitors. The assay in the microplate was performed using IPTG-induced cultures of *E. coli* HPD and *E. coli* EV, after reaching OD_600_ 0.6–0.8, mixed with TYR. In the HPD inhibitor assay wells, mesotrione, nitisinone and sulcotrione were added in a concentration range from 0 µM to 5 µM and tembotrione in a range from 0 µM to 20 µM with 4 replicates per concentration. After 24 h of incubation at 37 °C (900 rpm, 70% RH) the results previously achieved in the flasks and tubes were confirmed. Formation of the brown pyomelanin pigment was observed with *E. coli* HPD and supplementing one of the inhibitors decreased pyomelanin formation with increasing concentrations (Fig. 4a,b). A centrifugation step was added to avoid interference of the bacterial cells with the colorimetric quantification of the ochronotic pigment. The EV obtained an OD_405_ (OD_405-EV_) of 0.38 ± 0.04 and was used as a negative control. The percentage of inhibition was calculated following Eq. 1 and Eq. 2. The calibration curves were fitted by the sigmoidal logistic four-parameter equations and graphically visualised in Fig. 4b. The calculated IC_50_ values for each of the HPD inhibitors were 0.396 ± 0.048 µM (mesotrione), 0.244 ± 0.071 µM (nitisinone), 0.187 ± 0.037 µM (sulcotrione) and 0.610 ± 0.015 µM (tembotrione) as shown in Fig. 4c. These IC_50_ values illustrate that sulcotrione is the most potent human HPD inhibitor whereas tembotrione is the least potent inhibitor of the four tested compounds.

### Robustness of the assay

#### Plate uniformity and signal variability assessment

**Figure 5:**
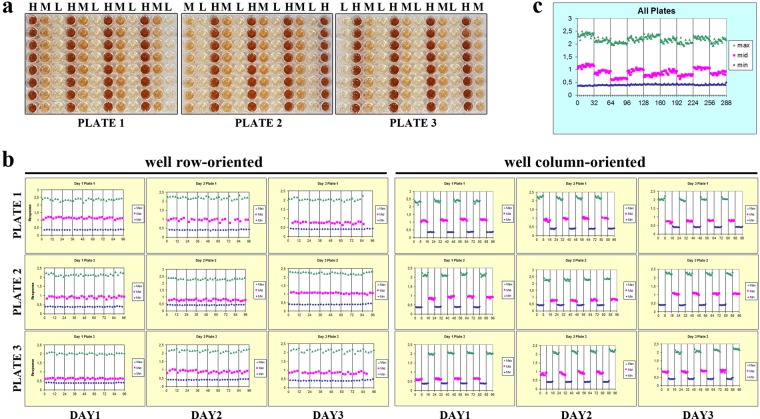
(**a**) To assess uniformity and separation of the signals of our new screening assay, a plate uniformity study was performed at maximum, medium and minimum signal −0%, 50% and 100% inhibition – with interleaved signal format. Different concentrations nitisinone were added to obtain a high (H), medium (M) and low (L) signal, i.e. 0-0.3-10 µM, respectively. We varied systematically the plate layout so that on every plate each signal was measured on a given day to obtain all signals on all plates while using an interleaved signal format (3 plates, 3 days). (**b**,**c**) A scatter plot also reveals patterns of drift or edge effect, where the signal is plotted against well number by row first, then by column. Here the plots of three plates run over three different days are shown and no edge effects or drift are observed.

New HTS assays should be validated for robustness of assay performance as well as for pharmacological relevance. To assess uniformity and separation of the signals of the new screening assay, a plate uniformity study was performed. The study was run over three days at the maximum (high), medium and minimum (low) signal or response levels to safeguard that the signal window is sufficient to detect inhibiting compounds (Fig. 5a,b). The raw signals must be sufficiently tight and a significant separation between the high and low signals to conduct screening is an overall requirement^29,30^. The maximum signal represented the readout signal in the absence of the inhibiting test compound, i.e. 0 µM nitisinone, the minimum signal measured the background signal or the maximally inhibiting concentration, i.e. 10 µM, and the medium signal estimated the variability at some point between the high and low signals, i.e. 0.3 µM (EC_50_). We varied systematically the plate layout so that on every plate each signal was measured on a given day to obtain all signals on all plates while using an interleaved signal format (Fig. 5a). The plate uniformity results in 96-well microplate format are represented in Table 1 and were used to calculate the CVs and Z′ factor (Eq. 1) values, taking into account 1 replicate per inhibitor or inhibitor concentration, in order to represent a real-life HTS assay. Maximum signal plate CVs were 1.82–4.06%, midpoint signal CVs were 2.74–7.56%, and minimum signal CVs were 2.28–5.86%. These CVs all pass the <20% criterion. Plate Z′ values were 0.87 ± 0.03 whilst the recommended acceptance criterion is Z′ factor ≥0.40 and related to the screening it can be seen as an excellent and robust assay^29^. The inter-plate tests, all within-day fold shifts <2 and all average (between-) day fold shifts <2 also meet the criteria.

**Table 1:** Results of the (intra) plate uniformity study. H, high signal; M; medium signal; L, low signal: CV, coefficient of variation; Mid.

Day	Plate	Type	Mean	SD	CV	Mid%	SW	Z′
1	1	H	2.38	0.07	3.12	61.25 ± 2.49	23.75	0.88
		M	1.15	0.05	4.36			
		L	0.37	0.01	2.28			
	2	H	2.15	0.06	2.76	70.11 ± 3.02	26.14	0.88
		M	0.91	0.05	5.82			
		L	0.39	0.01	3.01			
	3	H	2.04	0.04	1.82	85.13 ± 1.78	40.49	0.91
		M	0.64	0.03	4.62			
		L	0.39	0.01	2.51			
2	1	H	2.20	0.06	2.96	68.86 ± 3.94	23.90	0.87
		M	0.97	0.07	7.30			
		L	0.41	0.01	3.55			
	2	H	2.32	0.06	2.41	80.89 ± 2.66	30.12	0.89
		M	0.78	0.06	6.43			
		L	0.42	0.01	3.41			
	3	H	2.16	0.07	3.19	71.77 ± 3.93	21.59	0.85
		M	0.91	0.07	7.56			
		L	0.41	0.02	3.94			
3	1	H	2.05	0.07	3.20	78.74 ± 2.83	21.36	0.86
		M	0.76	0.05	6.06			
		L	0.42	0.01	3.03			
	2	H	2.25	0.05	2.40	64.13 ± 1.57	30.50	0.89
		M	1.06	0.03	2.74			
		L	0.40	0.02	3.87			
	3	H	2.12	0.09	4.06	73.96 ± 3.00	16.00	0.81
		M	0.86	0.05	5.98			
		L	0.41	0.02	5.86			

%, normalised mid signal; SW, signal window; Z′, Z′ factor. All max signal and all mid signal (unnormalised) CVs are <20%. All normalised mid signal (mid. %) SD’s <20, SW’s >2 and Z′ factors >0.4. All min (low) SD’s < Min (max (High) SD, mid SD). Z′ and CV values were calculated taking into account 1 replicate per compound or concentration, thereby mimicking a real-life HTS assay.

#### Spatial uniformity assessment

Due to evaporation during the incubation periods or plate stacking, plate edge or side effects can occur in HTS assays and need to be examined. Plates that do not exhibit material edge or drift effects are the overall requirement. Row and column drift were assessed using the high and medium signals from left-to-right and top-to-bottom (Fig. 5b). No drift effects that exceed 20% were observed, i.e. maximum drift observed for the high signal and medium signal was 6.23% and 13.79% resp., so material drift effects can be excluded. A scatter plot can also reveal patterns of drift or edge effect, where the signal is plotted against well number by row first, then by column (Fig. 5b,c). Figure 5b,c shows the plots of three plates run over three different days and no edge effects or drift were observed.

## Discussion

Human HPD is an imperative drug target that is involved in HT1 and AKU, yet a simple and robust HTS assay relying on human HPD is lacking in drug discovery and repositioning. To overcome this problem, we have developed an HTS colorimetric whole-cell bioassay for the fast identification and evaluation of new and human-specific HPD inhibitors as potential pharmacological treatment of TIMD. For this whole-cell bioassay we rely on the capability of recombinant *E. coli* that express human HPD, to generate a brown pigment, i.e. pyomelanin, after adding TYR.

Previously, a whole-cell colorimetric bioassay was developed using plant-derived HPD enzymes suitable for the development and the environmental risk assessment of new β-triketone herbicides. This assay not only allows to identify new plant HPD inhibitors, but also pan species-HPD inhibitors and was validated with high-performance liquid chromatography^18^. A major drawback of that assay is, however, that it is based on the use of the HPD enzyme of *Arabidopsis thaliana*, a plant-derived HPD enzyme that only has 34% sequence identity with the human HPD enzyme^18^. Although the assay allows to evaluate pan species-HPD enzyme inhibitors such as nitisinone, sulcotrione, mesotrione and tembotrione, it will not be useable for the future development of human-specific HPD inhibitors. Currently, no human-specific HPD enzyme inhibitors exist. Yet, the need for these human-specific HPD inhibitors is vast and desired over pan-species inhibitors such as nitisinone in order to decrease or even prevent some of the currently observed side-effects of HPD inhibition therapy in TIMD patients including metabolic perturbations in the patient’s microbiome^14^.

Besides the fact that this plant HPD-based assay is not human-specific, and thus cannot be used to identify human-specific HPD inhibitors, another major drawback relates to the recombinant HPD enzyme expression conditions, i.e. 4 °C and induction times that can take up to 48 hours or even 72 hours^18^. Induction at low temperature is widely used to increase the solubility of the desired recombinant proteins and to avoid aggregation. However, problems start to occur in the physiology of *E. coli* when cultivation temperatures below 10 °C–15 °C are used. These problems implicate interference with DNA, RNA, protein synthesis and fatty acid saturation^25,31,32^. In our study, an HTS assay using human HPD was developed solely relying on the conditions found in humans e.g. protein expression at 37 °C and physiological pH. To achieve this, a switch from the *E. coli* strain BL21 (DE3) to C43 (DE3) was required due to the observed toxicity of the foreign protein. Indeed, when the *E. coli* strain C43 (DE3) is induced, proliferation of intracellular membranes can occur which in fact contain overexpressed recombinant protein^33–35^. In addition, it is known that human HPD is present in the endoplasmic reticulum membrane and Golgi apparatus of human liver and kidney cells, so it is not surprising that human HPD was found to be present in the insoluble fraction of lysed *E. coli* C43 (DE3), bound to intracellular membranes, but still metabolically active. Using the *E. coli* strain C43 (DE3) did not only allow to work under human physiological conditions, but also significantly reduced the required assay time from 72 hours to 24 hours as compared to the previously reported plant HPD-based HTS assay^18^. As opposed to the previously reported plant HPD-based assay, the substrate L-tyrosine is added to the culture upon initiation of the protein expression, i.e. when adding IPTG. This facilitates *de novo* produced human HPD to immediately start metabolizing HPP after enzymatic conversion of L-tyrosine by *E. coli*’s tyrosine aminotransferases, and thus prevents problems that might occur due the degradation and/or instability of the human HPD enzyme when it is recombinantly expressed at 37 °C.

To allow high-throughput screening, our new bioassay was adapted to the 96-well microplate format. Previously reported assays in 96-well plate format to evaluate newly designed HPD inhibitors are all based on the coupled enzyme reaction of the isolated HPD enzyme with the crude extract of HGD and the formation of maleylacetoacetate measured at 318 nm^16,17,21,22^. Notwithstanding this cascade enzyme reaction is reliable, it is laborious and time-consuming as it involves the production and isolation of both enzymes on top of the assay itself and thus greatly depends on the quality of the HGD enzyme as well^16,17,21,22^. The use of a whole-cell bioassay opposed to isolated enzymes is desirable as these enzymes are more stable inside the whole cell, and secondly the need for exogenous co-factor addition, i.e. Fe (II), HEPES buffer and sodium ascorbate, is obsolete, making the whole cell assay very cost-effective^36–39^. In the same context, L-tyrosine was used as substrate instead of the much more expensive HPP. This is possible due to the natural presence of tyrosine aminotransferases (TAT) in *E. coli*, including aspC, tyrB and hisC^40–43^.

Oxygen is consumed upon conversion of HPP to HGA by the human HPD enzyme. A possible alternative to pyomelanin quantification could therefore be the measurement of the oxygen consumption rate as it was previously reported for the initial characterisation of dioxygenases. However, oxygen consumption cannot be used as an HTS parameter as interference of cell respiration in whole cells can occur^39^. In contrast, spectrophotometric quantification of pyomelanin formation at 405 nm allows a very specific and sensitive readout in a short measurement time^39^. As a result, our new whole-cell bioassay combines the advantages of *in situ* enzymatic conversion reactions with fast spectrophotometric analyses, leading to a reliable and robust HTS assay.

The validation of our whole-cell colorimetric bioassay, i.e. robustness testing according to the NCGC guidelines^30^, distinguishes our assay from all the previously reported HPD-based assays. For the other assays, the focus was mainly on making new inhibitors by rational design, whilst we developed a reliable, simple and particularly a robust assay. The Z′ value, a useful tool for the evaluation of the quality of an HTS assay, is 0.87 ± 0.03 in 96-well microplate format whilst the recommended acceptance criterion is Z′ factor ≥0.40. Consequently, this is considered as an excellent assay, especially since it was optimised manually and requires only 1 replicate per compound or concentration, making it truly high-throughput. We foresee that the throughput and robustness can even be further improved by miniaturising the assay to 384 and 1536 well-format using adjusted equipment including automatic pipetting systems and liquid-handling robotics.

Altogether, this study reports the establishment and validation of an HTS colorimetric whole-cell bioassay based on the recombinant expression of the human HPD enzyme for the fast identification and evaluation of novel human(-specific) HPD inhibitors as potential pharmacological treatment of TIMD.

## Methods

### Materials

All chemicals were of analytical reagent grade or higher quality and purchased from Sigma Aldrich Chemie GmbH (Schnelldorf, Germany), AppliChem (Darmstadt, Germany), or Carl Roth (Karlsruhe, Germany). Nitisinone was purchased from Yecuris (Tualatin, OR, USA). Microtiter plates (96-wells, v-shaped) for protein expression (Greiner Bio-one GmbH, Frickenhausen, Germany) were incubated in a Multitron II Infors Shaker (Infors AG, Bottmingen, Switzerland). For detection of absorbance Tecan Sunrise^®^ or Tecan Infinite^®^ M200 pro (Tecan Group Ltd., Männedorf, Switzerland) plate readers were used. Plasmid isolation kits were purchased from Macherey-Nagel GmbH & Co. (Düren, Germany).

### Bacterial strains, plasmids and target gene

*E. coli* DH5α (Stratagene, La Jolla, CA, USA) was used as a host for maintenance and propagation, whereas BL21 Gold (DE3) (Agilent Technologies, Santa Clara, CA, USA) and C43 (DE3) (Lucigen Corporation, Middleton, WI, USA) were used for expression. pET42b(+) from Novagen (Darmstadt, Germany) was used as a vector for high-level expression in *E. coli*, containing a gene for resistance to Kanamycin (KANA). A negative control, or empty vector (EV), was obtained with pET42b(+) plasmid introduction in *E. coli* and designated as EV. cDNA of wild type human HPD (NM_002150.2) was obtained from Geneart in a standard pMA-T-based cloning plasmid (Thermo Scientific), codon-optimised for *E. coli*., and subcloned using restriction enzymes and ligase-based cloning as an NdeI-XhoI fragment in the multiple cloning site (MCS) of pET42b(+) for high-level protein expression in *E. coli* bacteria. Lysogeny Broth (LB) was used as the standard medium supplemented with KANA (50 µg/mL) (LB_KANA_) to maintain selection pressure.

### Selection of the most optimal strain for expression

*E. coli* strains BL21 gold (DE3) and C43 (DE3) were compared to identify the most optimal strain for (over)expression of the human HPD enzyme. LB_KANA_- agar plates containing different concentrations IPTG, i.e. 0 mM, 0.5 mM and 1 mM, were inoculated with both strains after transformation (heat shock) with pET42b-HPD or EV. In addition, another set of LB_KANA_-agar plates with ITPG (0.5 mM and 1 mM) were supplemented with 0.75 mg/mL TYR and inoculated with both strains to check for HPD activity by the formation of pyomelanin from TYR. The agar plates were incubated overnight at 37 °C. Furthermore, LB_KANA_ medium containing 1 mM IPTG and 0.75 mg/mL TYR was inoculated with both *E. coli* strains in order to compare protein expression and activity. After 24 h of induction under shaking at 37 °C and 250 rpm (Multitron II; Infors GmbH, Einsbach, Germany), the OD_405_ was measured (Eppendorf Biophotometer Plus).

### Optimal expression conditions

For cell growth LB_KANA_ medium supplemented with 1% (m/v) glucose and a shaking incubator (Multitron II; Infors GmbH, Einsbach, Germany) was used for incubation (overnight, 37 °C, and 250 rpm). For expression, the overnight culture was diluted in fresh LB_KANA_ and incubated in a flask (30 °C, 250 rpm) up to an optical density at 600 nm (OD_600_) of 0.1 using a spectrophotometer (Eppendorf Biophotometer Plus). When OD_600_ reached 0.6–0.8, protein expression was induced with 1 mM IPTG. Various temperatures for optimal expression (37 °C and 30 °C) were assessed by Western Blot analysis and HPD activity times (0 h–48 h) were established by measuring OD_405_. Upon induction of HPD with IPTG, 0.75 mg/mL TYR and/or HPD inhibitor were added to the medium.

### Protein extraction, SDS-PAGE and western blot

For protein extract preparation, induced *E. coli* HPD cells were harvested and centrifuged (3000 g, 5 min, and 4 °C). The supernatant was discarded, the pellet was washed in 20 mM HEPES buffer, pH 7 (AppliChem, Darmstadt, Germany) and centrifuged (2400 g, 2 min) whereafter the supernatant was again discarded. The washed pellet was resuspended in 20 mM HEPES buffer, pH 7, cooled on ice and the *E. coli* HPD cells were disrupted by sonication at an amplitude of 40%, 3 × 30 seconds with an interval of 30 seconds. After sonication, supernatant and pellet were separated by centrifugation (maximum speed, 10 min, 4 °C) and both fractions (pellet resuspended in double distilled water) were analysed by western blot for protein expression pattern as previously described^44^. Briefly, proteins were separated on 12% Mini-Protean^®^ TGX Stain-free^TM^ gels (Bio-Rad, USA) using electrophoresis and blotted onto nitrocellulose Trans-Blot^®^ Turbo^TM^ Transfer Packs (Bio-Rad, USA) using a Trans-Blot^®^ Turbo^TM^ transfer system (Bio-Rad, USA). Subsequently, blocking of the membranes was carried out with blocking buffer (5% bovine serum albumin in Tris-buffered saline solution (i.e. 20 mM Tris – 135 mM NaCl) containing 0.1% Tween-20). The membranes were incubated overnight at 4 °C with primary antibody directed against human HPD, Monoclonal Anti-HPD antibody produced in mouse (Sigma-Aldrich, Saint-Louis, MO, USA), diluted 1:1000 in blocking buffer followed by 1-hour incubation at room temperature with polyclonal goat anti-mouse secondary antibody (Dako, Denmark), and diluted 1:1000 in blocking buffer. Excessive antibody was removed by washing the membranes three times in Tris-buffered saline solution with 0.1% Tween-20. Detection of HPD was performed by means of Pierce^TM^ enhanced chemiluminescence (ECL) Western Blotting substrate kit (Thermo Scientific, Rockford, IL, USA) and ChemiDoc MP imaging system (Bio-Rad, USA). Quantification of the obtained protein bands was accomplished using Bio-Rad ImageLab software (v.5.2.1). Pixel density data was normalized by subtracting the averaged pixel density of four background areas from the sample data.

### Colorimetric bioassay high throughput screening

For the colorimetric assay, recombinant *E. coli* HPD cells were induced with 1 mM IPTG, after reaching OD_600_ 0.6–0.8, in LB_KANA_ medium. Simultaneously, the medium was mixed with a volume of 7.5 mg/mL TYR and, if appropriate, a triketone inhibitor. After incubation (250 rpm in a Multitron II; Infors GmbH, Einsbach, Germany, 24 h, 37 °C, 70% RH), the culture was centrifuged (3200 g, 10 min) and OD_405_ of the supernatant was measured (Eppendorf Biophotometer Plus). For the HTS in 96-well MTP format, 200 µL of induced *E. coli* HPD cells, supplemented with 0.75 mg/mL TYR, were added to each well of a 96-well V-bottom expression MTP (Greiner bio-one GmbH, Frickenhausen, Germany). After incubation (900 rpm in a Multitron II; Infors GmbH, Einsbach, Germany, 24 h, 37 °C, 70% RH), the plate was centrifuged (3200 g, 10 min), and then 150 µL supernatant was transferred into 96-well flat bottom MTP (Greiner bio-one GmbH, Frickenhausen, Germany) for measurement at OD_405_ with an absorbance plate reader.

### Dose response inhibition curves

The colorimetric assay was challenged by a concentration range of mesotrione, nitisinone and sulcotrione (0–5 µM) and tembotrione (0–20 µM) with 4 replicates per concentration (n = 6). Protein expression and measurements were performed as described above. The normalised OD_405_ (OD_405-N_) was calculated according to

1 $${{\rm{OD}}}_{405 \mbox{-} {\rm{M}}}-{{\rm{OD}}}_{405 \mbox{-} {\rm{EV}}}$$

where OD_405-EV = _measured OD_405_ of the EV. The activity percentage, Act. %, was calculated according to the equation

2 $${\rm{Act.}} \% =({{\rm{OD}}}_{405 \mbox{-} {\rm{N}}}/{{\rm{OD}}}_{405 \mbox{-} {\rm{M}}})\ast 100$$

where OD_405-M_ is the mean of OD_405_ measured at 0 µM nitisinone (i.e. 100% activity). Being, % inhibition, Inh. %, 100 – Act. %. IC_50_ values for the HPD inhibitors were calculated with GraphPad Prism 7 software.

### Robustness of assay

A 3-day plate uniformity study at maximum, medium and minimum signal −0%, 50% and 100% inhibition – with interleaved signal format was run on separate days (3 days, 3 plates). To the expression culture, different concentrations nitisinone were added to obtain a high (H), medium (M) and low (L) signal, i.e. 0-0.3-10 µM, respectively. The same plate formats were used on all days of the test. The concentration of the medium signal, i.e. 0.3 µM, was not changed over the course of the test. A volume of 200 µL bacterial culture was added to each well of, respectively, a 96-well (Greiner bio-one GmbH, Frickenhausen, Germany) expression MTP according to a certain plate layout (plate 1: HML, plate 2: MLH, plate 3: LHM). Protein expression and measurements were performed as mentioned above. The mean, standard deviation, coefficient of variation (CV), signal window (SW), and Z′ factor were calculated. The Z′ factor was calculated according to the following equation taking into account 1 replicate per compound or concentration in future HTS applications.

3 $$Z^{\prime} =\frac{(AV{G}_{Max}-3\frac{S{D}_{Max}}{\sqrt{n}})-(AV{G}_{Min}+3\frac{S{D}_{Min}}{\sqrt{n}})}{AV{G}_{Max}-AV{G}_{Min}}$$

## Supplementary information

Supplementary Figure 1

## Data Availability

All data generated or analysed during this study are included in this published article.
