# Supplementary material for: A robust bacterial assay for high-throughput screening of human 4-hydroxyphenylpyruvate dioxygenase inhibitors
Source: Sci Rep. 2019 Oct 2;9:14145. doi: 10.1038/s41598-019-50533-1 (PMC6775094; doi:10.1038/s41598-019-50533-1)

A robust bacterial assay for high-throughput screening of human 4-hydroxyphenylpyruvate dioxygenase inhibitors

Jessie Neuckermans[1], Alan Mertens[2], Dinja De Win[1], Ulrich Schwaneberg[2], Joery De Kock[1]

[1] Department of *In Vitro* Toxicology and Dermato-cosmetology, Faculty of Medicine and Pharmacy, Vrije Universiteit Brussel, Laarbeeklaan 103, B-1090 Brussels, Belgium.

[2] Lehrstuhl für Biotechnologie, RWTH Aachen University, Worringerweg 3, 52074 Aachen, Germany.

Supplementary Fig. 1. Western blot analyses of the pellet fraction were performed for human HPD production by C43 (DE3) at 30 °C and 37 °C between 0 and 48 h post induction (**a-b**). Here, the original uncropped blots are shown. At circa 45 kDa a prominent protein band was observed after IPTG induction in the pellet fraction for the two assessed temperatures as marked by the red rectangle. Western blot analysis showed that the human HPD enzyme, represented by the lower band at ~ 45 kDa, was already weakly expressed one hour after induction with 1 mM IPTG at 37 °C, but not at 30 °C. 4 hours after induction the protein was highly expressed at 37 °C, but only moderately at 30 °C.

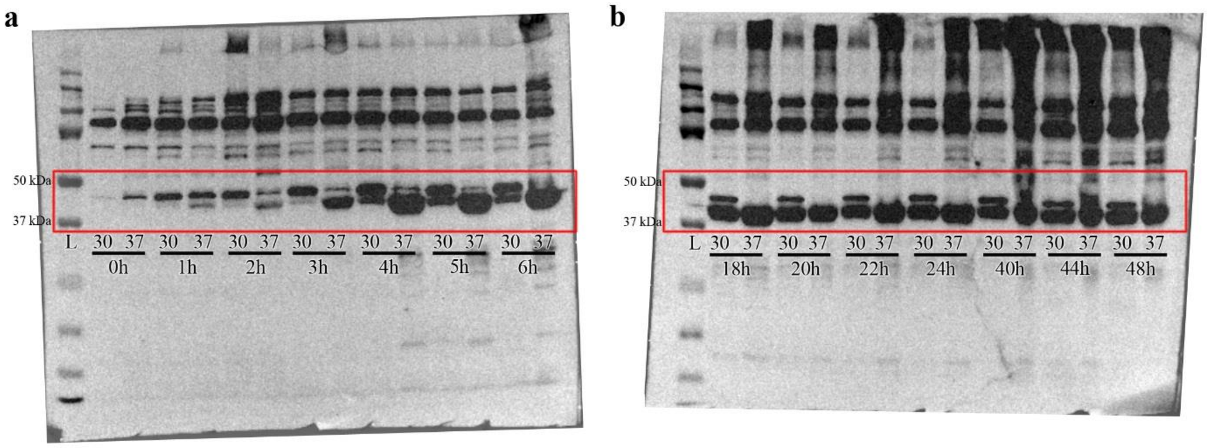

Supplement: Supplementary file 1 — Supplementary Figure 1 [file 41598_2019_50533_MOESM1_ESM.pdf]
